# Occurrence and Genetic Diversity of the Zoonotic Enteric Protozoans and *Enterocytozoon bieneusi* in Père David’s Deer (*Elaphurus davidianus*) from Beijing, China

**DOI:** 10.3390/pathogens11111223

**Published:** 2022-10-23

**Authors:** Peiyang Zhang, Qingxun Zhang, Shuyi Han, Guohui Yuan, Jiade Bai, Hongxuan He

**Affiliations:** 1 National Research Center for Wildlife-Born Diseases, Institute of Zoology, Chinese Academy of Sciences, Beijing 100101, China; 2 Beijing Milu Ecological Research Center, Beijing 100076, China

**Keywords:** Père David’s deer, enteric protozoans, fungal, prevalence, genetic diversity, China

## Abstract

*Cryptosporidium* spp., *Blastocystis*, *Giardia duodenalis*, *Balantioides coli*, *Pentatrichomonas hominis*, and *Enterocytozoon bieneusi* are enteric protozoan parasites and fungal species in humans and animals. Père David’s deer is an endangered species in China, but the prevalence of enteric protozoans in this species still needs to be further studied. Thus, we investigated the prevalence and genetic diversity of zoonotic parasites in Père David’s deer during the period of 2018–2021. Among the 286 fecal samples collected from Père David’s deer in the Nanhaizi Nature Reserve, 83 (29.0%) were positive for *Blastocystis*, 70 (24.5%) were positive for *E. bieneusi*, while other protozoan parasites were negative. Based on a phylogenetic analysis, three *Blastocystis* subtypes (ST10, ST14, and ST21) and ten *E. bieneusi* genotypes (Genotype D, MWC_d1, HLJD-V, Peru6, BEB6, BJED-I to BJED-I V) were identified. In addition, the *Blastocystis* subtype ST14 and the *E. bieneusi* genotype D and Peru6 were first detected in Père David’s deer. Our study first reports the presence of two enteric protozoans in Père David’s deer during a 4-year active surveillance and provides more information about zoonotic subtypes/genotypes of *Blastocystis* and *E. bieneusi* in deer.

## 1. Introduction

Parasitic protists and fungal species, including *Cryptosporidium* spp., *Blastocystis*, *Giardia duodenalis*, *Balantioides coli*, *Pentatrichomonas hominis*, and *Enterocytozoon bieneusi*, can infect a broad spectrum of hosts, including humans, domestic animals, and wildlife [1,2,3,4]. These pathogens are mainly transmitted via the fecal–oral route through the consumption of contaminated food and water and are associated with profuse or chronic diarrhea in the host. Wildlife animals are recognized as an environmental reservoir of human pathogenic enteric pathogens [5,6,7]. Frequent contact with different hosts can promote the cross-species transmission of zoonotic enteric protozoans [8]. Recently, molecular epidemiological studies have identified the common enteric protozoans and fungal species in captive and wild deer [9,10,11,12,13,14,15]. Several zoonotic species/genotypes of *Cryptosporidium* spp. and *E. bieneusi,* including *C. parvum*, *C. hominis*, *E. bieneusi* genotype D and IV, were previously detected in cervids [11,12]. More than ten *Blastocystis* subtypes have been identified in cervids, including zoonotic ST1, ST3, ST4, and ST10 [13]; four genetic assemblages of *G. duodenalis*, including zoonotic assemblage A and B and animal-specific assemblage D and E, are occasionally found in cervids [14]. *B. coli* and *P. hominis*, which are zoonotic but usually neglected protozoa, have undergone host range expansion in ruminants in the recent past [15,16]. To date, *B. coli* has been identified in ruminants (camels, cattle, sheep, and goat), while *P. hominis* infection has also been detected in sika deer from China.

Père David’s deer (*Elaphurus davidianus*) is presently classified as a Class I National Key Protected Species of China. It was extirpated in the wild in China at the end of the 19th century. Fortunately, 38 Père David’s deer were reintroduced to China from Britain in 1985 and were raised in the Nanhaizi Nature Reserve [17]. With the government taking steps toward population rejuvenation (three major populations), ex situ conservation, and a reintroduction into the wild, the population has reached more than 9000 individuals in 2021, with at least 2800 living in the wild [17]. However, there is little information on the occurrence and genetic diversity of enteric protozoan parasites in Père David’s deer. In the present study, we further investigated the molecular epidemiology, genetic diversity, and temporal dynamics of enteric protozoans in free-ranging Père David’s deer in Beijing, China. These findings could expand the host range of enteric protozoans and improve our understanding of the ecological distribution characteristics of zoonotic parasitic diseases.

## 2. Materials and Methods

### 2.1. Specimens Collection

From August 2018 to December 2021, a total of 286 fresh fecal specimens were collected from Père David’s deer in the Nanhaizi Nature Reserve in Beijing, China. The sample numbers for 2018, 2019, 2020, and 2021 were 42, 26, 96, and 122, respectively. Fresh specimens were immediately collected using individual polyethylene gloves and were transported to the laboratory in an ice box and stored in a sterile cryopreservation tube at −80 °C before DNA extraction.

### 2.2. DNA Extraction and PCR Amplification

Total DNA was extracted from each fecal sample (200 mg) using a TIANamp Stool DNA Kit (TIANGEN BIOTECH, Beijing, China), following the manufacturer’s instruction. The extracted DNA was stored at −20 °C for further PCR analysis. For the detection of *Blastocystis* and *E. bieneusi*, previously described nested PCR assays were used to amplify the 18S rRNA gene and the internal transcribed spacer (ITS), respectively. *Cryptosporidium* spp. were identified using nested PCR that amplified partial SSU rRNA and the 60 kDa glycoprotein (gp60) gene. *Giardia duodenalis* was identified by nested PCR, which amplified the partial SSU rRNA and the beta giardin (bg) gene. *P. hominis* was identified by the targeting of the ITS and 18S rRNA gene by nested PCR. The presence of *B. coli* was determined by using conventional PCR to target the ITS1-5.8S rRNA-ITS2 gene region of rRNA. All primers used in this study are listed in Table 1. The secondary PCR products were visualized on a 1.0% agarose gel using a UV transilluminator.

### 2.3. Sequencing and Phylogenetic Analysis

Positive products were sent to BGI Sequencing (BGI, Beijing, China) for sequencing. The sequences were assembled using the Seqman 7.1.0 software. The nucleotide sequences and reference sequences from the NCBI database were aligned by ClustalX to identify the genotypes. The SSU rRNA gene sequence of *Blastocystis* and the ITS sequences of *E. bieneusi* in this study as well as the representative sequences available in the GenBank database were used for phylogenetic analyses. A phylogenetic tree was constructed with MEGA 10.0 using the neighbor-joining (NJ) method in the Kimura 2-parameter model with 1000 bootstrap replicates.

### 2.4. Statistical Analysis

The variations in *Blastocystis* and *E. bieneusi* prevalence from different years were calculated by chi-squared test using SPSS 25.0. The results were considered statistically significant when *p* < 0.05.

## 3. Results and Discussion

### 3.1. Prevalence of Enteric Protozoans

A total of 286 fecal specimens from Père David’s deer were analyzed using nested/conventional PCR assays targeting six pathogens. The most prevalent protozoan parasite and fungus was *Blastocystis*, with a prevalence of 29.0% (83/286), followed by *E. bieneusi* (24.5%, 70/286) (Table 2). The remaining parasites were not detected at all. For *Blastocystis*, the infection rate in the period of 2018–2021 was 33.3% (14/42), 23.1% (6/26), 24.0% (23/96), and 33.6% (41/122), respectively. No significant difference between the years were observed (*p* > 0.05). In view of the molecular epidemiological research on *Blastocystis* in more than ten deer species worldwide, the infection rate ranged considerably from 6.7% to 88.8% [2]. The prevalence of *Blastocystis* observed in the present study was lower than those in white-tailed deer (88.8%, 71/80) in the USA [13], in Yezo sika deer (45.5%, 60/132) in Japan [26], and in water deer (40.8%, 51/125) in Korea [27]. However, the current findings were much higher than the *Blastocystis* prevalence in red deer (2.0%, 1/50) in Australia [28], in spotted deer (3.3%, 1/30) in Bangladesh [29], in sika deer (0.8%, 6/760) in northern China [30], and in sika deer (14.6%, 12/82) and reindeer (6.7%, 7/104) in several provinces of China [31]. To our knowledge, there was only one previous report of *Blastocystis* infection in Père David’s deer (56.3%, 72/128) in Hubei, China [32], which was much higher than our study. The significant difference in prevalence indicates that the different breeds and living conditions of Père David’s deer might have different sensitivities to *Blastocystis* [32,33].

The overall positive rate of *E. bieneusi* was 24.5% (70/286), and the infection rate in 2018–2021 was 42.9% (18/42), 34.6% (9/26), 9.4% (9/96), and 27.9% (34/122), respectively (Table 2). Similarly, the prevalence here was correlated with 35.2% (45/128) in Père David’s deer in Hubei [33], 34.0% (16/47) in Henan [34], and 30.0% (24/80) in Beijing [9]. Notably, the prevalence during the four years ranged from 9.4 to 42.9%, and the difference was statistically significant (*p* < 0.01), which may have been caused by differences in the season in which the specimens were collected. The samples from 2018 and 2019 were collected in summer, samples from 2021 were collected in spring and winter, while samples from 2020 were collected in winter. Our previous research on Père David’s deer parasites identified that gastrointestinal parasite prevalence, burden, and diversity were at their highest in summer and their lowest in winter [35]. The high protozoa prevalence is correlated with wetter seasons [36], which means that during summer, the deer may have greater exposure to protozoal pathogens. Typically, winters are drier than summers in Beijing Milu park, which might limit the transmission of protozoa between different individuals. Moreover, the farrowing and mating period for Père David’s deer peaks between March and September (wetter season), resulting in increased contact between females and newborns, both males and females. This increase in activity might have resulted in more chances for parasite transmission during the wetter seasons [7]. A more accurate analysis of the different seasons may yield further information on the temporal dynamics of *E. bieneusi* in Père David’s deer.

Four protozoa parasites, including *Cryptosporidium* spp., *G. duodenalis*, *B. coli*, and *P. hominis,* are common zoonotic infectious agents and pose a risk to public health. Although these pathogens were negative in the present study, some of these pathogens were commonly found in ruminants [37]. Previous studies have evidenced *Cryptosporidium* spp. infection in Père David’s deer, although at a low prevalence [33,38]. In addition, the current increase in the amount of information regarding the presence of *G. duodenalis*, *B. coli*, and *P. hominis* in deer might be due to the increased awareness of the rising zoonotic potential of these parasites [15,39,40,41].

### 3.2. Molecular Typing and Phylogenetic Analysis of Enteric Protozoans

**Figure 1:**
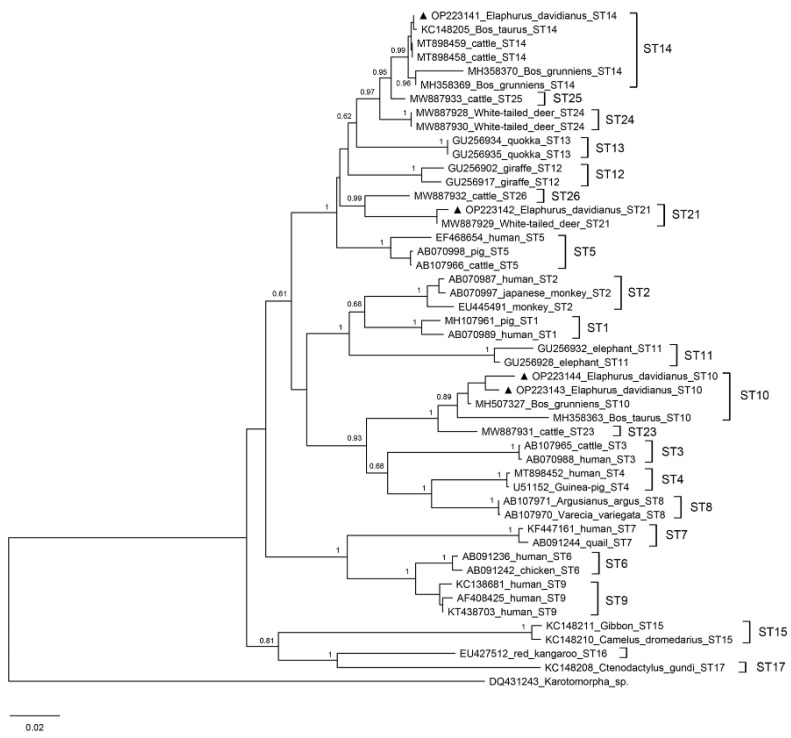
Phylogenetic reconstruction with the SSU rRNA nucleotide sequences of *Blastocystis* sp. obtained in this study and reference subtypes. The subtypes identified in this study are marked with a ▲ and highlighted in bold.

According to the SSU rRNA gene sequence analysis, three *Blastocystis* subtypes (ST10, ST14, and ST21) were identified in our study, with ST10 being the predominant subtype (48.8%, 41/84), followed by ST14 (42.9%, 36/84) and ST21 (8.3%, 7/84) (Table 2). Phylogenetic analysis implied that the isolates could be classified into animal-specific subtypes (ST10, ST14, and ST21), which shows limited zoonotic potential (Figure 1). According to a recent report on Père David’s deer in Hubei, five *Blastocystis* subtypes (ST10, ST21, ST23, ST25, and ST26) had been identified, with ST21 being the most prevalent subtype [32]. ST10 and ST14 have been commonly detected in pigs, horses, and ruminants such as cattle, sheep, goats, and deer [42,43]. ST21 seems specific to ruminants (cattle, goats, sheep, and deer), according to the previous studies [13,44,45].

**Figure 2:**
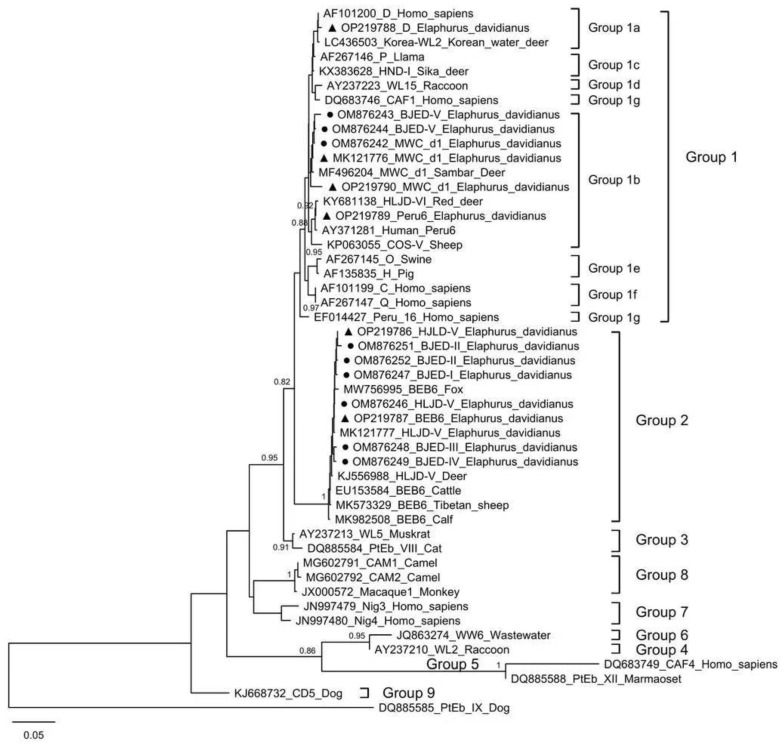
Phylogenetic tree of *E. bieneusi* genotypes identified in this study, and known genotypes based on the neighbor-joining analysis of the internally transcribed spacer of the rRNA gene. The new genotypes identified in this study are marked with a ▲ and highlighted in bold. Other genotype sequences identified in this study that are identical to those in our previous studies are marked with a ● and highlighted in bold.

Our study identified 10 distinct *E. bieneusi* genotypes (D, MWC_d1, HLJD-V, Peru6, BEB6, BJED-I to BJED-V) by ITS sequence analysis, which were classified into zoonotic Group 1 and Group 2. Genotype D belongs to subgroup 1a; MWC_d1, Peru6, and BJED-V belong to subgroup 1b; HLJD-V, BEB6, and BJED-I-IV belong to Group 2 (Figure 2). In our study, genotypes HLJD-V and MWC_d1 were the most prevalent genotypes in Père David’s deer, which was similar to the results of previous studies [9,33]. On the contrary, the genotypes carried by Père David’s deer in Henan are rather different (Type IV, EbpC, EbpA, BEB6, COS-I, and COS-II) [34]. After a 4-year active surveillance of enteric protozoans, zoonotic Genotype D and Peru6 were first identified in Père David’s deer. Genotype D is known as the most prevalent zoonotic genotype and is widely distributed in humans and animals worldwide [36,46]. Peru6 is found in humans and many kinds of animals [47,48]. Our previous study identified five new genotypes (BJED-I to BJED-V) in Père David’s deer [9], which were proven to be widespread during our 4-year active surveillance in the present study. In addition, Genotype BEB6 is also commonly found in ruminants, including deer [36,46,49], and it has also been detected in humans [50], which shows its zoonotic transmission risk. Research has shown that *Blastocystis* and *E. bieneusi* can cause diarrheal disease in humans and ruminants [49,50]. However, in the related studies mentioned above, fecal samples that were mainly collected from healthy deer showed no clinical signs of illness at the time of sampling. These results indicate that deer could serve as reservoirs and could play a role in the transmission of those pathogens between wildlife and humans or domestic animals.

## 4. Conclusions

In conclusion, our study broadens the knowledge on enteric protozoans and *E. bieneusi* infections in Père David’s deer from Beijing. Three *Blastocystis* subtypes and ten *E. bieneusi* genotypes were identified. Our study first reports the *Blastocystis* subtype ST14 and zoonotic *E. bieneusi* genotype D and Peru6 in Père David’s deer. The occurrence of zoonotic genotypes in *E. bieneusi* suggests that the deer may serve as a potential source of infection in human populations.

## Figures and Tables

**Table 1:** Primers for enteric protozoan parasites used in this study.

Pathogens	Target Gene	Primer Names	Sequence	Reference
*Blastocystis*	18S rRNA	RD3	GGGATCCTGATCCTTCCGCAGGTTCACCTAC	[18]
		RD5	GGAAGCTTATCTGGTTGATCCTGCCAGTA	
		BlF	GGAGGTAGTGACAATAAATC	
		BlR	CGTTCATGATGAACAATTAC	
*E. bieneusi*	ITS	EBITS3	GGTCATAGGGATGAAGAG	[9]
		EBITS4	TTCGAGTTCTTTCGCGCTC	
		EBITS1	GCTCTGAATATCTATGGCT	
		EBITS2.4	ATCGCCGACGGATCCAAGTG	
*Cryptosporidium* spp.	SSU rRNA	XF2f	GGAAGGGTTGTATTTATTAGATAAAG	[19]
		XF2r	AAGGAGTAAGGAACAACCTCCA	
		pSSUf	AAAGCTCGTAGTTGGATTTCTGTT	
		pSSUr	ACCTCTGACTGTTAAATACRAATGC	
	gp60	18S-F1	TTTACCCACACATCTGTAGCGTCG	[20]
		18S-R1	ACGGACGGAATGATGTATCTGA	
		18S-F2	ATAGGTGATAATTAGTCAGTCTTTAAT	
		18S-R2	TCCAAAAGCGGCTGAGTCAGCATC	
*Giardia duodenalis*	bg	G7	AAGCCCGACGACCTCACCCGCAGTGC	[21]
		G759	GAGGCCGCCCTGGATCTTCGAGACGAC	
		2005F	GAACGAACGAGATCGAGGTCCG	
		2005R	CTCGACGAGCTTCGTGTT	
	SSU rRNA	Gia2029F	AAGTGTGGTGCAGACGGACTC	[22]
		Gia2150c	CTGCTGCCGTCCTTGGATGT	
		RH11	CATCCGGTCGATCCTGCC	
		RH4	AGTCGAACCCTGATTCTCCGCCCAGG	
*Balantidium coli*	ITS1-5.8S rRNA-ITS2	B5D	GCTCCTACCGATACCGGGT	[23]
		B5RC	GCGGGTCATCTTACTTGATTTC	
*Pentatrichomonas hominis*	18S rRNA	Ph1	ATGGCGAGTGGTGGAATA	[24]
		Ph2	CCCAACTACGCTAAGGATT	
		Ph3	TGTAAACGATGCCGACAGAG	
		Ph5	CAACACTGAAGCCAATGCGAGC	
	ITS	ITS-F1	CGGTAGGTGAACCTGCCGTT	[25]
		ITS-R1	TGCTTCAGTTCAGCGGGTCT	
		ITS-F2	GGTGAACCTGCCGTTGGATC	
		ITS-R2	TTCAGTTCAGCGGGTCTTCC	

**Table 2:** Prevalence and genotype/subtype distribution of *E. bieneusi* and *Blastocystis* infection in Père David’s deer in Beijing, China.

Year	No. of Samples	*Blastocystis*		*Enterocytozoon bieneusi*	
		No. of Positive (%)	ITS Genotypes (No.)	No. of Positive (%)	Subtype (No.)
2018	42	14 (33.3)	ST10 (*n* = 5), ST14 (*n* = 4), ST21 (*n* = 5)	18 (42.9)	HLJD-V (*n* = 11),MWC_d1 (*n* = 2), Peru6 (*n* = 1), BJED-I (*n* = 1), BJED-II (*n* = 1), BJED-IV (*n* = 1), BJED-V (*n* = 1)
2019	26	6 (23.1)	ST10 (*n* = 2), ST14 (*n* = 4)	9 (34.6)	HLJD-V (*n* = 6), MWC_d1 (*n* = 1), BJED-II (*n* = 1), BJED-III (*n* = 1)
2020	96	23 (24.0)	ST10 (*n* = 7), ST14 (*n* = 16)	9 (9.4)	HLJD-V (*n* = 4), MWC_d1 (*n* = 1), D (*n* = 2), BEB6 (*n* = 2)
2021	122	41 (33.6)	ST10 (*n* = 27), ST14 (*n* = 12), ST21 (*n* = 2)	34 (27.9)	HLJD-V (*n* = 14), MWC_d1 (*n*= 10), BEB6 (*n* = 1), BJED-I (*n* = 1), BJED-II (*n* = 3), BJED-III (*n* = 1), BJED-IV (*n* = 1), BJED-V (*n* = 3)
Total	286	83 (29.0)	ST10 (*n* = 41), ST14 (*n* = 36), ST21 (*n* = 7)	70 (24.5)	HLJD-V (*n* = 35), MWC_d1 (*n* = 14), BEB6 (*n* = 3), D (*n* = 2), Peru6 (*n* = 1), BJED-I (*n* = 2), BJED-II (*n* = 5), BJED-III (*n* = 2), BJED-IV (*n* = 2), BJED-V (*n* = 4)

## Data Availability

The datasets presented in this study can be found in the article.
